# The combined effect of coating treatments to nisin, nano-silica, and chitosan on oxidation processes of stored button mushrooms at 4 °C

**DOI:** 10.1038/s41598-021-85610-x

**Published:** 2021-03-16

**Authors:** Rokayya Sami, Abeer Elhakem, Mona Alharbi, Nada Benajiba, Mohammad Fikry, Mahmoud Helal

**Affiliations:** 1 Department of Food Science and Nutrition, College of Sciences, Taif University, P.O. 11099, Taif, 21944 Saudi Arabia; 2 Department of Biology, College of Science and Humanities in Al-Kharj, Prince Sattam Bin Abdulaziz University, Al-Kharj, 11942 Saudi Arabia; 3 Department of Basic Health Sciences, Deanship of Preparatory Year, Princess Nourah Bint Abdulrahman University, P.O. Box 84428, Riyadh, 11671 Saudi Arabia; 4 Department of Agricultural and Biosystems Engineering, Faculty of Agriculture, Benha University, Moshtohor, Toukh, 13736 Qalyoubia Governorate Egypt; 5 Department of Mechanical Engineering, Faculty of Engineering, Taif University, P.O. 11099, Taif, 21944 Saudi Arabia

**Keywords:** Biochemistry, Biological techniques, Biotechnology, Plant sciences, Nanoscience and technology

## Abstract

*Agaricus bisporus* is an edible fungus with a limited shelf life due to high moisture loss, browning, respiration, self-dissolve, lack of physical protection, rotting, and microbial attack. Mushrooms have been coated with nisin, nano-silica, and chitosan films in order to extend the shelf life, preserve quality and oxidation activities. The results showed that treating the mushrooms with chitosan and nano-silica (CH-AN-SILICA) increased superoxide dismutase activity (SOD—6.53 U kg^−1^), total phenolic content (TPC—0.39 g kg^−1^), and malondialdehyde content (MDA—1.63 µmol kg^−1^). CH-AN-SILICA treatment exhibited the highest scavenging against 2,2-Diphenyl-1-picrylhydrazyl (DPPH) and 2,2′-azino-bis(3-ethylbenzothiazoline-6-sulfonic acid) (ABTS) radicals. While, CH-AN-SILICA with the addition of nisin as an antimicrobial agent preserved almost the reactive oxygen species such as hydroxyl radicals (OH—0.33 µmol g^−1^), superoxide anion (O_2_^•−^—0.271 mmol s^−1^ kg^−1^), and hydrogen peroxide (H_2_O_2_—21.54 µmol g^−1^). Besides, both CH-AN-SILICA and CH-AN-SILICA/N enhanced the catalase (CAT) activity and reduced the respiration rate. The results indicated that the combination of nisin, nano-silica, and chitosan coating films was effective in providing a longer storage life with acceptable quality in mushrooms.

## Introduction

Button mushrooms (*Agaricus bisporus*) are abundantly popular edible mushrooms with a significant commercial potential due to their valuable nutrients including proteins, main amino acids, riboflavin, and niacin. Furthermore, the valuable pharmacological, organoleptic, therapeutic bioactive compounds, antibacterial, immunomodulation, and antioxidant effects were noticed^1^. Though, that mushrooms have a limited shelf-life (1–3) days at the commercial temperature, due to several factors such as moisture loss, browning, respiration, self-dissolve, lack of physical protection rotting, and the microbial attack^2^. Browning, active metabolic process, and rapid deterioration especially with the white strains are important indexes that affect the marketing and customer acceptability^3^. Oxidation enzymes play an essential role in catalyzing the phenols hydroxylation to form *o*-diphenols and then *o*-quinones, which negatively influence the browning index, cellular membrane structure, and malondialdehyde content^4^. Besides, reactive oxygen species balance can be destroyed during the storage period followed by low enzymatic antioxidant defense such as superoxide dismutase, peroxidase, and catalase. At present, a variety of preservation methods have been technically performed to delay browning and extend the shelf-life of mushrooms such as chemical washing^5^, UV irradiation^6^, the modified atmosphere^7^, chilling^8^, and biological treatments^9^. On the other hand, there were some side effects for these techniques, such as the potential toxicity, sensory evaluation, and lack of nutrients^10^. Consequently, it is crucial to find out an eco-friendly preservation method to extend the storage time and maintain the quality of mushrooms. Qu et al.^11^, have proposed essential oils due to their antimicrobial effects against the lipophilic properties of the mushroom cells. In recent years, nano-technology coating has been reported for the antioxidant capacities potentially enhancement for several types of foods. Qiao et al.^12^, have proposed nano-coating, thymol, and tween-80 as antimicrobial agents to delay the oxidation processes on cantaloupes. Sami et al.^13^, applied nano-coating, chitosan, and nisin for the inhibition of polyphenol oxidase and peroxidase activities on blueberries. The American Food and Drug Administration (FDA) had declared that nano-silica dioxide, less than 2% by weight of the food is safe to be used as a food additive in food preservation and industry^14^. Limited studies have been described the effects of coating treatments on the oxidation processes of button mushrooms during postharvest storage. Thus, due to the limited shelf-life of mushrooms, it is needed to focus on the biochemical changes such as respiration, and production rates with the measurement of bioactive components and antioxidant activities, reactive oxygen species, and malondialdehyde contents to prolong the shelf-life.

## Results

### Effect of coating on oxidative enzyme activities

Table 1 presents the effect of coating on oxidative enzyme activities. Peroxidase activity (POD) results during storage, raised to the greatest activity on the 9th day, and then hurriedly reduced. However, CH-AN-SILICA/N treatment (0.005 U kg^−1^) significantly (*p* < 0.05) inhibited POD activity during the thorough storage period that might avoid the melanin synthesis at the mushroom browning process. Superoxide dismutase activity (SOD) in treated mushrooms radically increased and existed higher than Control/M samples, (Table 1). CH-AN-SILICA/N reported the highest value of SOD activity (4.15 U kg^−1^). CAT activity of mushroom samples coated with CH-AN-SILICA and CH-AN-SILICA/N films reported equal values (0.47 U kg^−1^). Mushrooms treated with CH-AN film only reported (0.44 U kg^−1^) lower CAT activity after 12 days of storage (*p* < 0.05).

**Table 1:** Effect of coating on oxidative enzyme activities; POD, SOD, and CAT in (Ukg^−1^).

	Days	Control/M	CH-AN	CH-AN-SILICA	CH-AN-SILICA/N
POD (Ukg^−1^)	0	0.003 ± 0.000^e^	0.003 ± 0.000^e^	0.003 ± 0.000^e^	0.003 ± 0.000^e^
	3	0.005 ± 0.001^d^	0.006 ± 0.001^d^	0.007 ± 0.001^d^	0.007 ± 0.001^d^
	6	0.007 ± 0.002^b^	0.007 ± 0.003^b^	0.009 ± 0.001^b^	0.009 ± 0.003^b^
	9	0.011 ± 0.001^a^	0.010 ± 0.001^a^	0.010 ± 0.003^a^	0.010 ± 0.002^a^
	12	0.009 ± 0.003^c^	0.007 ± 0.002^c^	0.006 ± 0.002^c^	0.005 ± 0.001^c^
SOD (Ukg^−1^)	0	3.323 ± 0.006^c^	3.323 ± 0.006^e^	3.323 ± 0.006^d^	3.323 ± 0.006^d^
	3	3.383 ± 0.007^b^	3.550 ± 0.010^d^	3.617 ± 0.006^c^	3.627 ± 0.006^c^
	6	3.417 ± 0.005^a^	3.593 ± 0.006^c^	3.703 ± 0.007^b^	3.667 ± 0.004^bc^
	9	3.423 ± 0.004^a^	3.613 ± 0.005^b^	3.717 ± 0.004^b^	3.837 ± 0.001^b^
	12	3.413 ± 0.006^a^	3.700 ± 0.010^a^	4.103 ± 0.032^a^	4.150 ± 0.002^a^
CAT (Ukg^−1^)	0	0.224 ± 0.007^b^	0.224 ± 0.007^b^	0.224 ± 0.006^b^	0.224 ± 0.005^b^
	3	0.325 ± 0.001^ab^	0.352 ± 0.002^ab^	0.391 ± 0.001^a^	0.381 ± 0.002^a^
	6	0.391 ± 0.001^a^	0.461 ± 0.001^a^	0.450 ± 0.001^a^	0.421 ± 0.001^a^
	9	0.381 ± 0.002^a^	0.440 ± 0.001^a^	0.491 ± 0.002^a^	0.441 ± 0.002^a^
	12	0.331 ± 0.001^ab^	0.435 ± 0.001^a^	0.471 ± 0.001^a^	0.469 ± 0.001^a^

Values within a column (lowercase) are significantly different (*p* ≥ 0.05). The values in parentheses indicate (SD ±) standard deviation.

### Effect of coating on TPC and MDA

**Figure 1:**
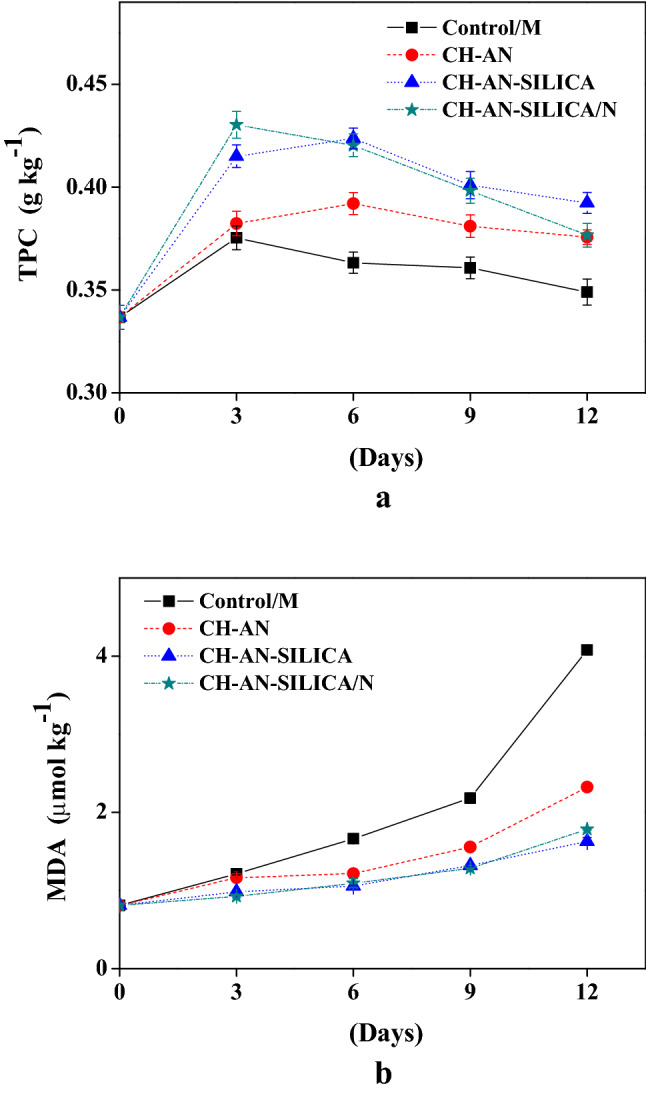
Effect of coating on (TPC, g kg^−1^) (**a**) and (MDA, µmol kg^−1^) (**b**).

Figure 1a presents the total phenolic content converted into nisin, nano-silica, and chitosan in the mushroom samples depending on the storage period. The initial TPC in fresh mushroom samples was counted (0.34 g kg^−1^). As a result, the increase of that consideration was minor, reaching (0.38–0.43 g kg^−1^) on the first 3 days of the storage, directly after applying the coatings. The highest statistically significant TPC rise was reported in the mushroom samples earlier subjected to CH-AN-SILICA coating at the end of the storage time. The MDA values in both Control/M and CH-AN mushrooms increased during the storage days as presented in Fig. 1b. MDA increased from 0.81 to 4.08 µmol kg^−1^ for Control/M, and from 0.81 to 1.63 µmol kg^−1^ for CH-AN-SILICA treated mushrooms.

### Effect of coating on respiration rate

**Figure 2:**
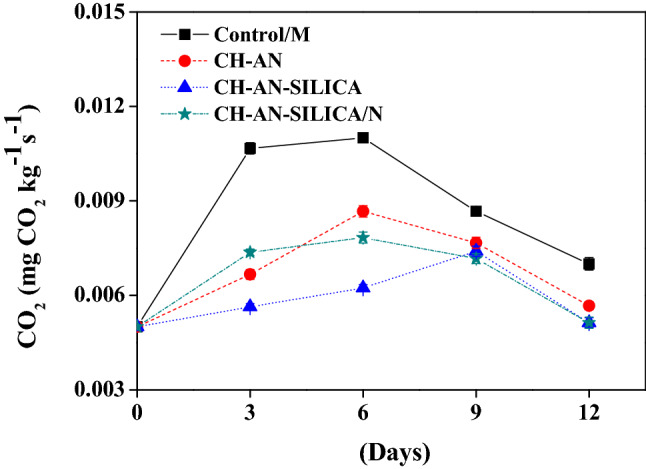
Effect of coating on respiration rate in (mg CO_2_ kg^−1^ s^−1^).

Figure 2 shows the respiration rate changes during storage after various coating treatments on button mushroom samples. The respiration rate was first raised and then reduced during the storage period. The mushrooms treated with CH-AN-SILICA or even CH-AN-SILICA/N films reported the lowest value (0.005 mg CO_2_ kg^−1^ s^−1^) at the end of 12 days of the storage period.

### Effect of coating on antioxidant capacity

**Figure 3:**
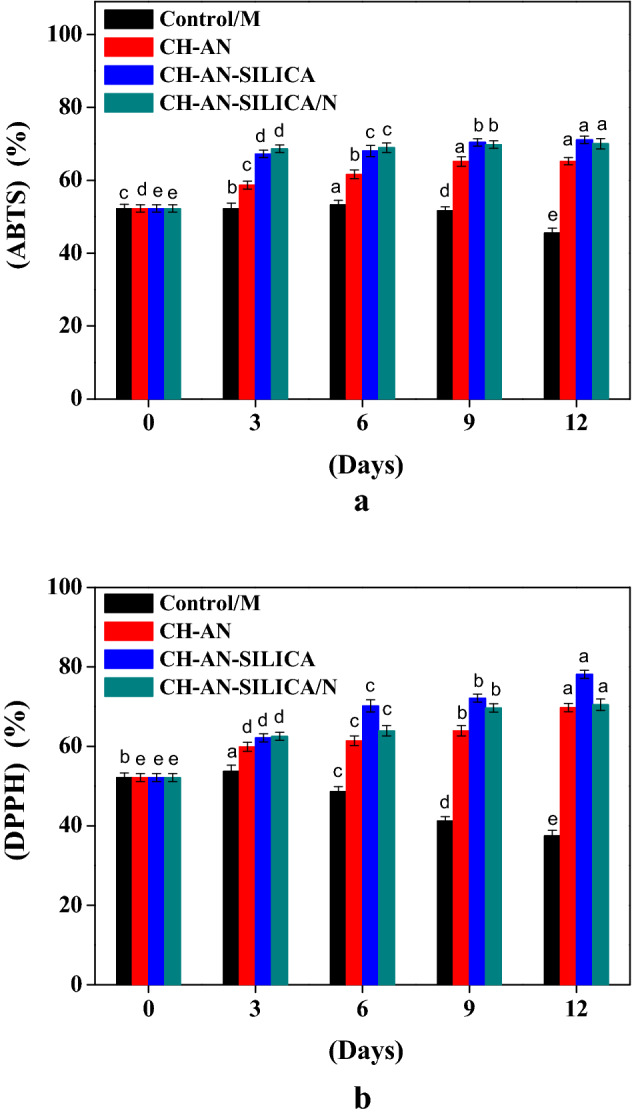
Effect of coating on antioxidant capacity; (ABTS, %) (**a**), (DPPH, %) (**b**).

ABTS and DPPH radical scavenging capacities in Control/M and treated mushrooms are presented in Fig. 3. In the current work, the initial antioxidant capacities immediately following the harvest were 52.26 and 52.14%, respectively. The results for ABTS radical scavenging increased and then declined at the end of the storage to reach a level of (71.09 and 70.03%) for CH-AN-SILICA and CH-AN-SILICA/N treatments, respectively, Fig. 3a. The DPPH radical scavenging capacity of treated mushroom samples was evaluated and compared to Control/M samples. CH-AN-SILICA treatment exhibited the highest scavenging against (DPPH) radicals (78.13%), Fig. 3b. However, the Control/M scavenging activity on the same day was 37.49%.

### Effect of coating on OH, O_2_^•−^, and H_2_O_2_

**Figure 4:**
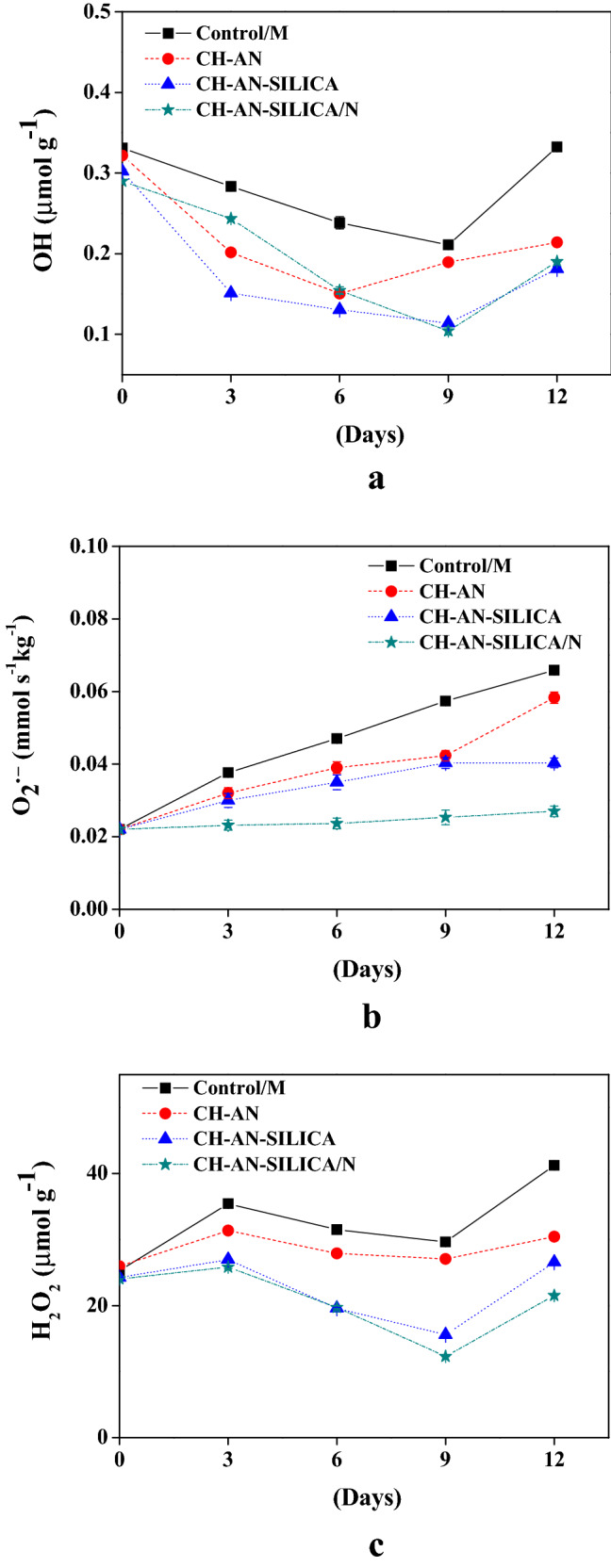
Effect of coating on reactive oxygen species;(OH, µmol g^−1^) (**a**), (O_2_^•−^ , mmol s^−1^ kg^−1^) (**b**), and (H_2_O_2_, µmol g^−1^) (**c**).

OH values were differentiated between the coating treatments at the end of the storage time (0.18–0.33 µmol g^−1^), where the lowest values were detected in (CH-AN-SILICA) and (CH-AN-SILICA/N) films, Fig. 4a. The O_2_^•−^ production rate harshly increases during the storage period from (0.022–0.271 mmol s^−1^ kg^−1^) in Control/M samples, Fig. 4b. The augmentation of O_2_^•−^ production rate was clear in the first 3 days for all the groups then declined after 6 days of the storage period. Among all the treatments, mushroom samples coated with CH-AN-SILICA/N reported the lowest O_2_^•−^ production rate and greatly inhibited the increase of O_2_^•−^ production rate in mushroom samples. H_2_O_2_ is responsible for the mushroom senescence and the destruction of cell integrity^15^. The H_2_O_2_ levels in mushroom samples treated with various coating treatments and stored for 12 days are shown in Fig. 4c. The results showed that there was a great increase in H_2_O_2_ values, especially in Control/M samples during storage. CH-AN-SILICA/N treatment followed by CH-AN-SILICA and CH-AN films reduced as compared to Control/M which were nearly doubled (41.24 µmol g-1).

## Discussion

POD, SOD, and CAT were evaluated during cold storage to control the oxidative stress balance caused by coating treatments to nisin, nano-silica, and chitosan. The quality characteristics were beginning to deteriorate after 3rd day due to oxidative enzymes which may perhaps change the color, odor, and taste.POD enzyme can associate with the browning index of a variety of fruits and vegetables^4^. Browning can be influenced by the oxidation of the phenolic substance and result in forming a brown-colored pigment as POD activity can hasten the browning reactions^16^. Moreover, the coating film with the addition of nisin can fill the groove of the POD active site between hydrogen bonds and Π–sigma, or even Π–Π interactions^13^. The current results are in agreement with Karimiradet al.^2^, who reported that POD activity had lower values after the oil coating treatments compared to the control, which might be due to minor oxidative stress retardant enzymatic discolorations. High SOD activity levels can reduce the free radical aggregation by H_2_O_2_ formation as the superfluous H_2_O_2_ should convert into non-toxic molecules^17^. In the case of CAT activity, it may be able to decrease the oxidative damage caused by H_2_O_2_ production in mushrooms^18^. Ding et al.^1^, reported a similar tendency for CAT activity on treated mushrooms. In comparison, nano-coating with the combination of chitosan components can prolong the mushroom's shelf-life up to 12 days. In the current study, the application of nano-coating with the combination of chitosan components partially inhibited the oxidative enzyme activities (POD, SOD, and CAT) of mushroom samples due to forming a protective barrier on the mushroom surface and reducing the oxygen supply.

TPC expressed in gallic acid equivalents agreed to these findings^19,20^ which reported that *Agaricus bisporus* has effective antioxidant activity and can be considered as original natural antioxidants. Eissa^21^, reported TPC changes in button mushrooms during storage of 15 days in cold conditions. The maximum increase was observed on the 3rd day, and then a decrease until the end of the storage period. That can be related to the physiological reaction damage during storage and exposure to oxygen.MDA is generally created by the peroxidation of lipid of the cell membrane as a direct indicator of the membrane injury^4^. The MDA values represented that the coating considerably improved the membrane lipid peroxidations. Therefore, the nano-coating with the combination of chitosan component delayed mushroom senescence and quality deterioration by minimizing the oxidative injury of button mushrooms during the whole storage period.

Respiration occupies the oxidation of energy-rich organic molecules such as organic acids, sugar, and starch into simpler molecules such as carbon dioxide and water with energy production^22^. While the high rate of respiration is associated with the quality loss of the samples. Gholamiet al.^23^, reported similar results for respiration rate which might be an indicator for the spoilage of mushroom samples. Those results indicated that the coating films were extremely efficient in decreasing the mushroom respiration rates.

CH-AN-SILICA treatment exhibited the highest scavenging against (DPPH) and (ABTS) radicals through enhancing TPC and motivating oxidation activities in treated samples leads to a rise in the antioxidant capacity of mushrooms. Sami et al.^24^, reported that nano-films coatings can efficiently decrease microbial load contamination, deterioration, improve postharvest quality, and prolong the shelf life of white button mushrooms during storage.

Reactive oxygen species mention the oxygen metabolites with high chemical reactivity, such as OH, O_2_^•−^, and H_2_O_2_, which are considered as toxic in the plant metabolism, which destroys the cytoplasmic membrane, causes senescence and lipid damage^25^. OH produced in the human body plays a vital role in tissue injury at sites of inflammation and oxidation for several diseases^26^. The current results declared that coating treatment maintained the lowest hydroxyl radicals activities by the inhibition of hydroxyl radicals of the methanol extract during the storage days. Ding et al.^1^, reported a decrease in O_2_^•−^ production rate was detected in mushroom samples treated with the 4-methoxy cinnamic acid. CH-AN-SILICA with the addition of nisin as an antimicrobial agent preserved almost the reactive oxygen species such as (OH—0.33 µmol g^−1^), (O_2_^•−^—0.271 mmol s^−1^ kg^−1^), and (H_2_O_2_—21.54 µmol g^−1^).

Coating the mushrooms with nisin, nano-silica, and chitosan films had useful effects on the quality and oxidation activities. The results of the current work showed that coatings, especially with nano-silica, were environmentally friendly treatment and efficient in reducing oxidation activities, respiration, and reactive oxygen species during cold storage.

## Materials and methods

### Material

Fresh button mushrooms used in this research were carefully selected from the science college laboratory at Taif University, in Taif City. A total number of 160 mushroom caps (forty caps in each basket, three baskets in each group). Mushrooms were in the maturity stage, uniform size, with wholly closed caps, intact, and with no sign of spoilage. They were pre-cooled at 4 ± 1 °C and a humidity of 65 ± 1% for 24-h to prevent the growth before the experiment evaluations.

### Preparation of films and coating processes

Chitosan powder (85%), acetic acid, nisin, and nano-silica (15 nm) were obtained from Sigma Aldrich Company, USA. Chitosan (CH-AN) as the main coating film was prepared by blending 1 g in distilled water plus acetic acid 1% at (pH = 4) and homogenized overnight until (pH = 5.5). While CH-AN-SILICA film was prepared by blending 1% nano-silica in CH-AN solution and CH-AN-SILICA/N film was prepared by blending 1% of nisin as a safe food additive. The dipping process was applied to coat the mushrooms. Control/M group was dipped well with distilled water for 1 min, while other samples were soaked into the coating films and then dried for 2 h by an electric fan to lose excess moisture at the ambient temperature. The coated samples were separately packed a zipped lock polyethylene bags and were stored at the cold condition of 4 °C^24^. Packaged samples were evaluated every 3 days up to 12 days.

### Extractions and oxidative enzyme activities assays

Oxidative enzyme activities such as peroxidase (POD), superoxide dismutase (SOD), and catalase (CAT) were evaluated on mushroom samples during storage. Approximately 15 g of each group were homogenized in 20 mL of 0.1 mol phosphate buffer (pH 7.0) and filtered to remove debris from the cells^27^. The supernatants were centrifuged at 6000 r/min for 15 min at 4 °C and collected for enzyme activities determinations as follows.

POD activity was determined by the procedure of Bi et al.^28^ The mixture consisted of 7 mL of 0.1 mol L^−1^ (pH = 5.5) sodium acetate monoacetate buffer, 3 mL of 25 mmol L^−1^ guaiacols, and 0.5 mL mushroom supernatants. The absorbance was recorded at 470 nm after 15 s as an initial value and recorded continuously every 30 s until 3 min.

SOD activity was evaluated by the procedure of Li et al.^29^ The mixture contained 1 mL of enzyme extract, 0.05 mol L^−1^ K-phosphate buffer (pH = 7.8), 0.2 mL of 750 mol L^−1^ nitro-blue tetrazolium, 0.2 mL of 130 mmol L^−1^ methionine0.2 mL of 100 μmol L^−1^, 0.1 mL of mmol L^−1^ riboflavin and placed under 4000 l × irradiance at 25 °C for 1 h. The absorbance was evaluated at 560 nm and expressed in U kg^−1^.

CAT activity was evaluated by the procedure of Liu et al.^30^ The mixture contained 0.1 mL of enzyme extract, 1 mL of 0.05 mol L L^−1^ sodium phosphate buffer (pH = 7.8), and 1 mL of 0.2% H2O2. The absorbance was evaluated at 240 nm at an interval of 30 s for 3 min and expressed in U kg^−1^.

### Determination of total phenolic content (TPC) and malondialdehyde content (MDA)

TPC was evaluated according to the method by Sami et al.^31^ In general, 80% ethanol was blended with 5 g of each group and centrifuged at 12,000 r/min for 20 min at 4 °C. Approximately 2 mL of 7% sodium carbonate was blended with 0.8 mL of collected supernatant and reacted with 1 mL of Folin–Ciocalteu reagent. The absorbance was evaluated at 760 nm and gallic acid was used as a standard for the qualifications.

MDA was measured as described by Sami et al.^4^ Approximately 3 g of each group were blended with 10 mL of 10% trichloroacetic acid (TCA) and centrifuged at 12,000 r/min for 20 min at 4 °C. The supernatant was mixed with 2 mL of 0.5% 2-thiobarbituric acid (TBA), boiled for 15 min, cooled, and evaluated at 450, 532 and 600 nm, respectively.

### Respiration rate evaluation

The respiration rate of the mushrooms was detected with a handheld three-gas analyzer (F950, FELIX, USA)^18^. Four groups of mushroom samples were put in a confined space in the containers. Two needle-sized holes have been opened in the containers, sealed to avoid the leakage of air, and kept at 4 °C for 4 h. Consequently, CO_2_ volume percentage was evaluated in triplicate according to the following formula:

$${\text{Respiration}}\;{\text{rate}}\;\left( {{\text{mg}}\;{\text{CO}}_{2} \;{\text{kg}}^{ - 1} {\text{s}}^{ - 1} } \right) = \frac{{\left( {\Delta y_{{{\text{CO}}_{2} }} \times V} \right)}}{{\left( {100 \times W \times \Delta t} \right)}}$$

where Δ*y*CO_2_ is the concentration fraction increment (%), *V* (mL) is the free volume, *W* (kg) is the mushroom weight, and Δ*t* (s) is the testing time in seconds.

### Determination of antioxidant capacity

2,2-Azino-bis-(3-ethylbenzothiazoline)-6-sulphonate activity (ABTS) was determined according to the mentioned method by Elhakem et al.^32,33^, while 2,2-diphenyl-1-picryhydrazyl activity (DPPH) of treated mushrooms was evaluated by using the method^34–36^ ABTS and DPPH activities in methanol (MeOH) were calculated as a percentage after measuring at 414 and 515 nm, respectively.

### Determination of hydroxyl radicals (OH), superoxide anion (O_2_^•−^), and hydrogen peroxide (H_2_O_2_)

The hydroxyl radical activity of button mushrooms extract was evaluated by the method of Dhanabalan et al.^26^ Stock solution 100 μL of 1 mM EDTA was prepared in DMSO and 10 μL of 10 mM ferric chloride, 100 μL of 1 mM ascorbic acid, 100 μL of 10 mM H_2_O_2_, 360 μL 10 mMdeoxyribose, and 330 μL of 50 mM phosphate buffer (pH = 7.4) were prepared in distilled water. Following incubation for 1 h at 37 °C, 1 mL of the mixture was added to the reaction solution containing 1 mL of 10% tricyclic antidepressant and 1 mL of 0.5% thiobarbituric acid, and 1 mL of 0.025 M NaOH. The absorbance was evaluated at 532 nm and expressed as μmol g^−1^.

The rate of O_2_^•−^ production was evacuated according to a commercial kit purchased from (Solarbio, China, Beijing) against NaNO_2_ as a standard curve previously described by Oyetayo^37^. Approximately 5 g of mushroom tissues were homogenized with 5 mL of phosphate buffer, 1 mL *p*-aminophenylsulfonic acid, 1 mL α-naphthylamine and centrifuged at 12,000 r/min for 20 min at 4 °C. The supernatant was evaluated at 550 nm and expressed as mmol s^−1^ kg^−1^.

The hydrogen peroxide content was evaluated according to the method by Song et al.^17^Approximately 5 g of mushroom samples were blended with 30 mL of acetone ((CH_3_)2CO), and centrifuged at 10,000 r/min for 15 min at 4 °C. HCL (including 200 mL L^−1^ titanium tetrachloride), and ammonia (17 mol L^−1^) were added to the mushroom supernatant then was washed with ((CH_3_)2CO) and dissolved in 2 mol L^−1^ H_2_SO_4_. The absorbance was measured at 410 nm and expressed as μmol g^−1^.

### Statistical analysis

All the current experiments were conducted on three replicates for each treatment. A total of forty white button mushroom caps for each treatment were used. One-way ANOVA test version 8.2 (SAS, Cary, NC, USA) was performed to compare the means at the significance level of *p* < 0.05.
